# Cellullar insights into cerebral cortical development: focusing on the locomotion mode of neuronal migration

**DOI:** 10.3389/fncel.2015.00394

**Published:** 2015-10-07

**Authors:** Takeshi Kawauchi

**Affiliations:** ^1^Department of Physiology, Keio University School of MedicineTokyo, Japan; ^2^Precursory Research for Embryonic Science and Technology (PRESTO), Japan Science and Technology AgencySaitama, Japan; ^3^Laboratory of Molecular Life Science, Institute of Biomedical Research and Innovation, Foundation for Biomedical Research and InnovationKobe, Japan

**Keywords:** microtubule, actin cytoskeleton, endocytosis, JNK, Rab5, Rab11, Rab7, Rap1

## Abstract

The mammalian brain consists of numerous compartments that are closely connected with each other via neural networks, comprising the basis of higher order brain functions. The highly specialized structure originates from simple pseudostratified neuroepithelium-derived neural progenitors located near the ventricle. A long journey by neurons from the ventricular side is essential for the formation of a sophisticated brain structure, including a mammalian-specific six-layered cerebral cortex. Neuronal migration consists of several contiguous steps, but the locomotion mode comprises a large part of the migration. The locomoting neurons exhibit unique features; a radial glial fiber-dependent migration requiring the endocytic recycling of N-cadherin and a neuron-specific migration mode with dilation/swelling formation that requires the actin and microtubule organization possibly regulated by cyclin-dependent kinase 5 (Cdk5), Dcx, p27^kip1^, Rac1, and POSH. Here I will introduce the roles of various cellular events, such as cytoskeletal organization, cell adhesion, and membrane trafficking, in the regulation of the neuronal migration, with particular focus on the locomotion mode.

## Introduction

**Figure 1:**
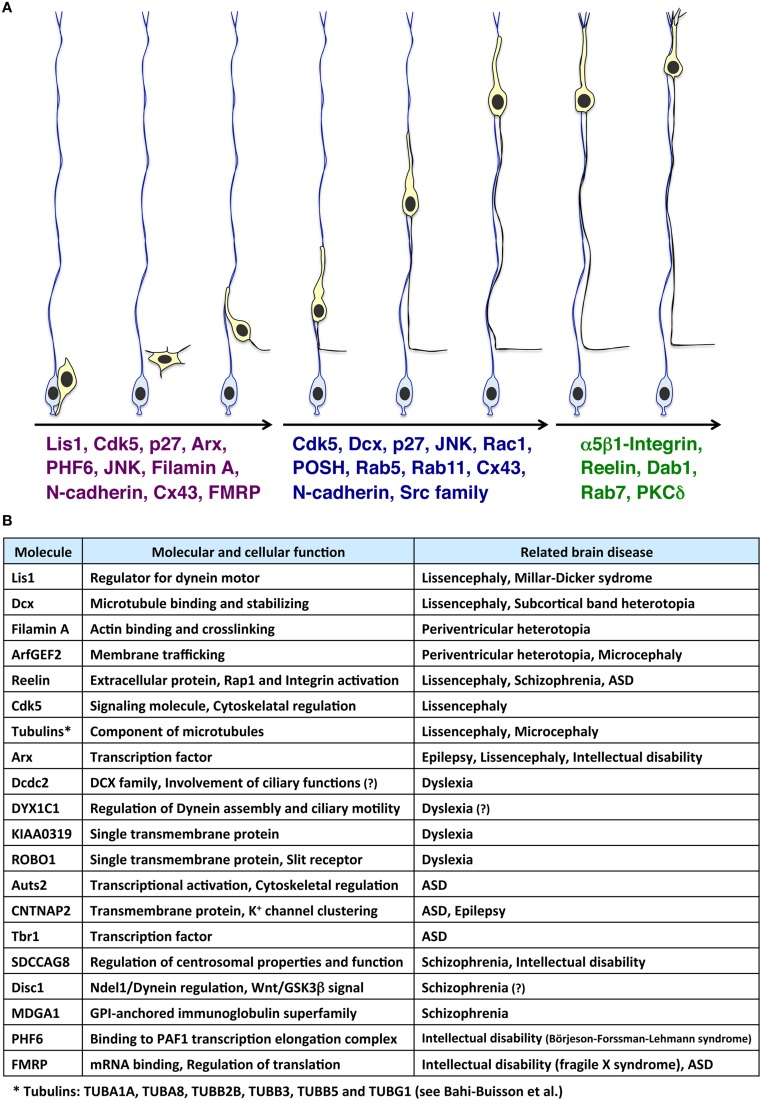
**Molecules involved in the multi-step modes of neuronal migration and its related neurological disorders**. **(A)** Immature neurons (light yellow cells), generated from radial glial progenitors (light blue cells) near the ventricle, migrate toward the pial surface. At the early phase of neuronal migration (the left three migrating neurons), many cytoskeletal-regulatory proteins (e.g., Lis1 and Filamin A), kinases (e.g., Cdk5 and JNK), and other proteins (e.g., p27^*kip*1^ and N-cadherin) are required for proper morphological changes of migrating neurons (Kawauchi and Hoshino, 2008; Lickiss et al., 2012; Kawauchi, 2014). Interestingly, some of these molecules are also involved in neurological disorders, such as Börjeson-Forssman-Lehmann syndrome (for PHF6) (Zhang et al., 2013a; Franzoni et al., 2015), Fragile X syndrome (for FMRP) (La Fata et al., 2014) and lissencephaly (for Lis1, Cdk5, and Arx) (Friocourt and Parnavelas, 2010; Reiner and Sapir, 2013; Magen et al., 2015). Subsequently, neurons undergo the locomotion mode of neuronal migration (the middle three migrating neurons). Cdk5 and its substrates, Dcx, and p27^kip1^, control the dilation/swelling formation during the locomotion. POSH and Rac1 are also required for the dilation/swelling formation. N-cadherin and its regulation by Rab5- and Rab11-dependent endocytic recycling play important roles in radial glial fiber-dependent migration of the locomoting neurons. At the final phase of migration, neurons undergo the terminal translocation mode (the right two migrating neurons). The Reelin-Dab1-C3G-Rap1-Talin-Integrin pathway regulates the terminal translocation. PKCδ is also required for the terminal translocation. **(B)** Molecules involved in neuronal migration-related brain diseases.

The brain is divided into many compartments, such as nuclei, layered structures, and cortical areas, allowing highly organized role allocations. The systematically allocated neuronal populations are generated from spatially restricted regions, the ventricular, and subventricular zones. Therefore, a long-distance migration from the ventricular side to the final destination is essential for constructing a functional brain. In line with this, defects in neuronal migration are associated with various neurological disorders (Gleeson and Walsh, 2000; Kawauchi and Hoshino, 2008). Several types of cortical malformations, including lissencephaly, double cortex syndrome (subcortical band heterotopia) and periventricular heterotopia (PVH), are thought to result from neuronal migration defects. These cortical malformations are frequently associated with intellectual disability and intractable epilepsy (Francis et al., 2006; Moon and Wynshaw-Boris, 2013; Reiner and Sapir, 2013; Lian and Sheen, 2015). *Lis1, Dcx, Filamin A, ArfGEF2, Arx, Reelin*, and several *Tubulin* genes (*TUBA1A, TUBA8, TUBB2B, TUBB3, TUBB5*, and *TUBG1)* are identified as causative genes for these cortical malformations (des Portes et al., 1998; Fox et al., 1998; Gleeson et al., 1998; Hong et al., 2000; Kitamura et al., 2002; Kato et al., 2004; Sheen et al., 2004; Keays et al., 2007; Abdollahi et al., 2009; Reiner and Sapir, 2013; Bahi-Buisson et al., 2014; Magen et al., 2015) (Figures 1A,B). Furthermore, suppression of genes related to dyslexia (e.g., *DCDC2, KIAA0319*), autism spectrum disorder (ASD) (e.g., *Auts2, CNTNAP2*) and schizophrenia (e.g., *SDCCAG8*) disturbs neuronal migration, although it is unclear whether the neuronal migration defect is the main cause of the pathogenesis of these neurological and psychiatric disorders (Hannula-Jouppi et al., 2005; Kamiya et al., 2005; Meng et al., 2005; Paracchini et al., 2006; Wang et al., 2006; Kähler et al., 2008; Peñagarikano et al., 2011; O'roak et al., 2012; Zhang et al., 2013a; Hori et al., 2014; Insolera et al., 2014; La Fata et al., 2014) (Figure 1B). Interestingly, in addition to these genes, several environmental factors, such as stress and inflammation, are also associated with cortical development, including neuronal migration (Stolp et al., 2012; Hashimoto-Torii et al., 2014; Ishii and Hashimoto-Torii, 2015).

**Figure 2:**
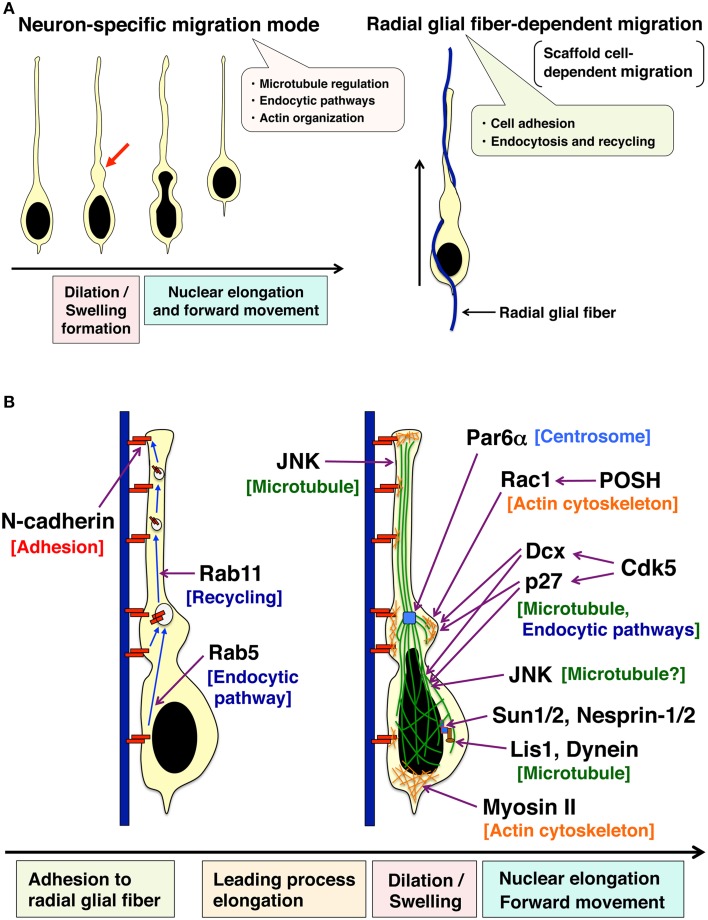
**Two unique features of the locomotion mode of neuronal migration**. **(A)** Locomoting neurons exhibit a neuron-specific migration feature (left) and a scaffold cell-dependent migration (right). Locomoting neurons form a cytoplasmic dilation/swelling at the proximal region of a leading process, and subsequently, the nucleus shows an elongated morphology and moves into the dilation/swelling (left). Locomoting neurons also show a scaffold cell-dependent migration. They attach to and migrate along the neural progenitor-derived radial glial fibers (right). **(B)** Cellular events regulating two unique features of the locomotion mode of neuronal migration. N-cadherin is involved in the adhesion to radial glial fibers. Endocytic recycling of N-cadherin is required for the forward movement of neurons. JNK controls the leading process formation possibly through the regulation of microtubule dynamics. Par6α is localized at centrosomes and regulates perinuclear cage-like microtubules. Both POSH-Rac1 and Cdk5-Dcx pathways are required for the formation of cytoplasmic dilation/swelling. Another Cdk5 substrate, p27^kip1^ also plays an important role in cytoplasmic dilation/swelling formation. Furthermore, Cdk5, Dcx, p27^kip1^ as well as JNK are involved in the nuclear elongation in the locomoting neurons. Lis, Dynein, SUN1/2, Nesprin-1/2, and Myosin II regulate the nuclear forward movement. See the main text for more details.

In the developing cerebral cortex, neuronal migration consists of several contiguous steps (Nadarajah and Parnavelas, 2002; Cooper, 2014; Takano et al., 2015) (Figure 1A). Newly generated neurons exhibit multipolar morphology in the lower part of the intermediate zone. The multipolar neurons form an axon and a leading process almost coincidentally with retraction of other processes. The bipolar-shaped neurons are called “locomoting neurons,” which migrate along radial glial fibers with unique morphological changes (the locomotion mode of neuronal migration) (Figure 2A). At the final phase of neuronal migration, they switch over from the “locomotion” into a “terminal translocation.”

Most studies so far have focused on the mechanisms of the morphological changes at the early phase of neuronal migration, including the multipolar-to-bipolar transition (Kawauchi and Hoshino, 2008; Heng et al., 2010). One reason is that many cortical malformation-related gene products are involved in the multipolar-to-bipolar transition. Second, the acquirement of neuronal polarity, a key step of neuronal maturation, occurs nearly simultaneously with the multipolar-bipolar transition. Third, suppression of cytoskeletal proteins or kinases often leads to defects in the early phase of migration due to the occurrence of various morphological changes at this stage.

In contrast to the early phase of migration, our knowledge of the locomotion mode is relatively poor. However, recent morphological and cell biological analyses have uncovered unique features of locomoting neurons. In this review, I will introduce recent advances in the molecular and cellular biology of neuronal migration with particular focus on the locomotion mode.

## The early phase of neuronal migration

Several steps of the early phase of neuronal migration, including the multipolar-to-bipolar transition, are required for the formation of the morphologies of the locomoting neurons. The formation of a leading process requires c-jun N-terminal kinase (JNK) (Kawauchi et al., 2003). Filamin A (FLNA), an actin-binding protein, is also involved in the early phase of migration, possibly including the leading process formation (Nagano et al., 2004). Knockdown of Lis1, a regulator of the dynein complex, a microtubule minus end-directed motor, suppresses the multipolar-bipolar transition (Tsai et al., 2005). FLNA and Lis1 have been identified as causative genes for PVH and lissencephaly, respectively, and both knockout of FLNA and Lis1 heterodeficiency show neuronal migration defects (Hirotsune et al., 1998; Zhang et al., 2013b).

In addition, many other molecules are reported to regulate the formation of multipolar morphology (e.g., Cdk5, p27^kip1^, Arx, Rab5) (Kawauchi et al., 2006, 2010; Friocourt et al., 2008; Friocourt and Parnavelas, 2010) and multipolar-to-bipolar transition (e.g., Cdk5, PHF6, FMRP) (Ohshima et al., 2007; Zhang et al., 2013a; La Fata et al., 2014; Franzoni et al., 2015) at the early phase of migration (Figure 1A).

## The locomotion mode of neuronal migration

The locomotion mode of neuronal migration covers the largest part of the neuronal journey, and is therefore a main contributor to proper neuronal positioning (Rakic, 2006; Nishimura et al., 2010). As described above, however, analysis of molecular mechanisms underlying the locomotion mode is difficult, because in many cases, neurons with defects in cytoskeletal proteins or kinases also show abnormalities early in neuronal migration prior to starting the locomotion (or no phenotypes). However, recent advances in *in vivo* cell biological approaches and novel technologies have uncovered several molecules regulating the unique features of the locomotion mode of neuronal migration. For example, a novel method, the *ex vivo* chemical inhibitor technique, that allows us to directly analyze molecules involved in the locomotion mode, has recently been established (Nishimura et al., 2010). Using this technique, Cdk5 and Src family kinases were shown to regulate the locomotion mode (Nishimura et al., 2010).

The locomotion mode of neuronal migration displays two major characteristics, a radial glial fiber-dependent migration and a neuron-specific unique migration mode with dilation/swelling formation and nuclear elongation (Rakic, 1972; Bellion et al., 2005; Schaar and McConnell, 2005) (Figure 2). In the next sub-sections, I will introduce the morphological, molecular, and cellular mechanisms of these unique characters of the locomotion mode.

### A unique migration mode with dilation/swelling formation

Locomoting neurons exhibit distinct migration features (Bellion et al., 2005; Schaar and McConnell, 2005; Nishimura et al., 2014). (1) Locomoting neurons extend a leading process and form a cytoplasmic dilation (also referred as to “swelling” especially in tangentially migrating interneurons) at the proximal region of a leading process. (2) The nucleus in the locomoting neurons becomes elongated to enter the cytoplasmic dilation (Figure 2).

The cytoplasmic dilation or swelling was first identified in 2005 as a migrating neuron-specific subcellular domain, because not only other migrating cells, such as neutrophils, keratocytes, and fibroblasts, but also static neurons do not form a cytoplasmic dilation/swelling (Bellion et al., 2005; Schaar and McConnell, 2005). Electron microscopy studies show that the cytoplasmic dilation/swelling contains the centrosome, Golgi apparatus, and microtubules. Although the centrosome frequently is a part of the cytoplasmic dilation/swelling (Bellion et al., 2005; Schaar and McConnell, 2005), suppression of dynein heavy chain or Lis1, both of which are known to regulate centrosomal positioning and nuclear forward movement in radially migrating neurons, does not disrupt cytoplasmic dilation/swelling (Tsai et al., 2007). Furthermore, mDia, an actin nucleator that acts as a downstream effector of RhoA, regulates centrosomal positioning, and nuclear translocation in tangentially migrating GABAergic interneurons. However, mDia deficiency does not impair the cytoplasmic dilation/swelling formation (Shinohara et al., 2012).

In contrast to RhoA, another Rho family small GTPase, Rac1 and its binding protein, POSH, are required for the formation of cytoplasmic dilation/swelling in cortical excitatory neurons (Yang et al., 2012). Suppression of Rac1 by the expression of the dominant negative mutant, shRNA-mediated knockdown, or gene targeting, disturbs neuronal migration (Kawauchi et al., 2003; Chen et al., 2007; Govek et al., 2011; Yang et al., 2012). Although Rac1 promotes the activity of JNK, which is known to regulate leading process morphology and neuronal migration (Kawauchi et al., 2003), JNK1-suppressing neurons are able to form the cytoplasmic dilation/swelling. The area of the cytoplasmic dilation/swelling is not significantly different between control and JNK1-knockdown neurons, although the morphologies of the cytoplasmic dilation/swelling in the JNK1-knockdown neurons are rough and irregular in part (Nishimura et al., 2014). Therefore, Rac1 and POSH are believed to control the formation of cytoplasmic dilation/swelling mainly in a JNK1-independent manner.

Considering that abundant microtubules are observed in the cytoplasmic dilation/swelling, microtubule-regulatory proteins may be involved in the formation of this subcellular domain. In fact, it has been reported that Dcx (previously known as Doublecortin) and its upstream kinase, Cdk5, are required for the formation of the cytoplasmic dilation/swelling (Nishimura et al., 2014). Interestingly, both *Dcx* and *Cdk5* are known as causative genes for lissencephaly (Gleeson et al., 1998; des Portes et al., 1998; Magen et al., 2015).

Dcx controls not only microtubule polymerization but also endocytic trafficking (Francis et al., 1999; Gleeson et al., 1999; Yap et al., 2012; Yap and Winckler, 2015), and clathrin-coated pits are observed in cytoplasmic dilation/swelling (Shieh et al., 2011). Pharmacological inhibition of microtubule polymerization (nocodazole treatment) or endocytosis (dynasore treatment) by using the *ex vivo* chemical inhibitor technique, disturbs the formation of the cytoplasmic dilation/swelling (Nishimura et al., 2014). Consistently, knockdown of Rab5, a regulator for endocytosis and trafficking to early endosomes, shows defects similar to the dynasore treatment.

Furthermore, p27^kip1^, another Cdk5 substrate, is required for the cytoplasmic dilation/swelling formation (Nishimura et al., 2014). Although p27^kip1^ controls the G1 length in the cell cycle and promotes cell cycle exit, p27^kip1^ also plays a role in actin reorganization through suppression of RhoA activity and activation of an actin-binding protein, Cofilin, in the multipolar processes of neurons at the early phase of migration (Kawauchi et al., 2006, 2013). In addition, p27^kip1^ is required for the tangential migration of cortical GABAergic interneurons via microtubule organization (Godin et al., 2012). However, it is still unclear which downstream event(s) (the regulation of actin or microtubule or something else) is important for the cytoplasmic dilation/swelling formation in the locomoting neurons.

### Nuclear elongation and forward movement

After the formation of the cytoplasmic dilation/swelling, the nucleus elongates, and moves into the newly formed dilation. This nuclear elongation is closely coupled with the cytoplasmic dilation/swelling formation. In fact, suppression of Cdk5, Dcx, p27^kip1^, Rab5, microtubule polymerization, or endocytosis perturbs the nuclear elongation as well as dilation/swelling formation during the locomotion mode (Nishimura et al., 2014). Interestingly, however, knockdown of JNK, which does not affect the area of the cytoplasmic dilation/swelling, suppresses the nuclear elongation, suggesting that Cdk5 and JNK, both of which promote microtubule dynamics (Kawauchi et al., 2003, 2005), have different roles in the locomotion mode of migration (Nishimura et al., 2014).

The elongated nuclei are surrounded by perinuclear cage-like microtubules, which contain abundant tyrosinated tubulins, components of dynamic microtubules (Rivas and Hatten, 1995; Schaar and McConnell, 2005; Umeshima et al., 2007). The regulation of microtubule dynamics is known to require Cdk5 and JNK activities (Kawauchi et al., 2005). Cdk5 phosphorylates focal adhesion kinase (FAK) at Ser732, and Ser732-phosphorylated FAK is localized on the perinuclear cage-like microtubules (Xie et al., 2003). Cdk5 deficiency or expression of the Ser732-nonphosphorylatable mutant of FAK (S732A) disturbs the nuclear elongation in migrating neurons. It is also known that overexpression of Par6α, which is localized at the centrosome, disrupts the perinuclear cage (Solecki et al., 2004).

The forward movement of the nuclei (nucleokinesis) requires Lis1- and dynein-mediated motor activity (see the following excellent reviews: Tsai and Gleeson, 2005; Marín et al., 2010). SUN1/2 and Nesprin-1/2, which are localized at the inner and outer membranes of the nuclear envelope, respectively, connect the nucleus to the dynein complex on microtubules in the locomoting neurons (Zhang et al., 2009). In addition, actomyosin-mediated contractility at the posterior end of the cell is known to play an important role in the nuclear forward movement (Schaar and McConnell, 2005; Martini and Valdeolmillos, 2010). Myosin II is also observed at the proximal region of the leading process and controls the coordinated movement of the centrosome and soma in cerebellar granule neurons (Solecki et al., 2009).

### A radial glial fiber-dependent migration

Another feature of the locomotion mode of neuronal migration is migration on other cells, called a scaffold cell-dependent migration (Kawauchi, 2012) (Figure 2A). It has been suggested that Astrotactin (Astn1) is involved in the interaction between migrating neurons and Bergmann glial fibers (Adams et al., 2002). Treatment with antibodies against Astn1, but not N-cadherin and L1-CAM, inhibits the attachment of cultured cerebellar granule neurons to astroglia and glia-guided neuronal migration (Stitt and Hatten, 1990; Fishell and Hatten, 1991).

The discovery that the locomoting neurons migrate along radial glial fibers in the developing cerebral cortex was reported in 1972 (Rakic, 1972). Unlike the cerebellar granule neurons, suppression of a cell-cell adhesion molecule, N-cadherin, in the developing cerebral cortex perturbs the attachment of migrating neurons to the radial glial fibers and neuronal migration (Kawauchi et al., 2010; Shikanai et al., 2011). Importantly, a portion of N-cadherin is internalized by Rab5-dependent endocytic pathways, and subsequently transported to the plasma membrane via Rab11-dependent recycling pathways. This active transport of N-cadherin is essential for the radial glial fiber-dependent migration of locomoting neurons in the developing cerebral cortex (Kawauchi et al., 2010).

N-cadherin is involved in other modes of neuronal migration and adhesions between radial glial neural progenitors (Solecki, 2012). Under the control of Reelin and Rap1, N-cadherin regulates the transition from multipolar to bipolar neurons (Jossin and Cooper, 2011; Gärtner et al., 2012). N-cadherin is also required for the somal translocation mode of neuronal migration, which is applied to the early-born neurons (Franco et al., 2011). Cajal-Retzius cells in the marginal zone and somal translocating neurons express immunoglobulin-like adhesion molecules, Nectin-1 and Nectin-3, respectively, and Nectin-3 upregulates N-cadherin to promote the somal translocation mode in early corticogenesis (Gil-Sanz et al., 2013). Interestingly, N-cadherin has also been implicated for a role in the tangential migration of cortical interneurons (Luccardini et al., 2013, 2015).

In addition to N-cadherin, other cell adhesion molecules, such as Connexin 43 (Cx43), Cx26, and JAM-C, have been shown to control neuronal migration. Cx43 and Cx26, gap junction proteins, stabilize a leading process on the radial glial fibers via enhancement of cell-cell adhesion, rather than formation of an aqueous channel (Elias et al., 2007). FAK promotes the assembly of Cx26 at contact sites between the locomoting neurons and radial glial fibers (Valiente et al., 2011). Interestingly, Cx43 is also involved in the formation of multipolar morphologies at the early phase of neuronal migration (Liu et al., 2012). Cx43 upregulates p27^kip1^, which controls the multipolar morphologies through actin reorganization (Kawauchi et al., 2006). JAM-C and its binding adaptor protein, Pard3, are localized at the tight junctions in epithelial cells. However, in migrating cerebellar granule neurons, Pard3A promotes the recruitment of JAM-C to neuron-neuron or neuron-glial cell contacts (Famulski et al., 2010).

Integrin heterodimers are mainly involved in cell-to-extra cellular matrix adhesion (Kawauchi, 2012). It has been reported that treatment with antibodies against β1-integrin suppresses radial glial fiber-dependent neuronal migration *in vitro* (Anton et al., 1999). However, NEX promoter-mediated conditional knockout of β1-integrin in neurons revealed no migration defects in the cortical six-layered structures, while Nestin promoter-mediated disruption of β1-integrin in both neurons and radial glial progenitors resulted in disorganization of radial glial fibers and cortical laminae, similar to type II-lissencephaly (Graus-Porta et al., 2001; Belvindrah et al., 2007).

## The final phase of neuronal migration after the locomotion: a terminal translocation mode

At the final phase of neuronal migration when the leading process reaches the marginal zone, neurons undergo a short-distance migration in a radial glial fiber-independent manner (Figure 1A). N-cadherin expression is decreased at the cell soma of neurons undergoing terminal translocation (Kawauchi et al., 2010). Suppression of Rab7, a regulator for lysosomal degradation pathways, leads to a defect in the terminal translocation. Taken together with the involvement of Rab7 in the degradation of N-cadherin *in vitro*, it suggests that Rab7-dependent lysosomal degradation of N-cadherin at the cell soma is required for the terminal translocation (Kawauchi et al., 2010). N-cadherin is still expressed in the distal region of the leading processes (immature dendrites) in terminal translocating neurons (Kawauchi et al., 2010), and therefore might play a role in the terminal translocation as it has been reported to control somal translocation during early corticogenesis (Franco et al., 2011).

Additionally, involvement of other cell adhesion molecules, such as α5β1-integrin, a receptor for fibronectin, and L1-CAM, and Protein kinase C delta (PKCδ) has been reported (Nishimura et al., 2010; Sekine et al., 2012; Tonosaki et al., 2014) (Figure 1A). Suppression of either α5-integrin or β1-integrin perturbs terminal translocation (Sekine et al., 2012). Reelin-mediated activation of Rap1 promotes the recruitment of Talin to the plasma membrane, which activates the Integrin heterodimers possibly through direct binding to the cytoplasmic region of β1-integrin (Sekine et al., 2012). As described above, Reelin also enhances the activation of Rap1 during the early phase of neuronal migration (Jossin and Cooper, 2011). Recent studies have revealed that two guanine-nucleotide exchange factors (GEFs), C3G, and RapGEF2, differentially activate Rap1 at the final or early phases of migration, respectively (Ye et al., 2014). However, defects in the multipolar-to-bipolar transition have been reported in C3G-knockout brains (Voss et al., 2008), suggesting that C3G may also be required for the early phase of neuronal migration.

## Conclusion

From the 1990's, several key molecules involved in neuronal migration, such as Lis1, Dcx, FLNA, and Reelin, have been identified mainly by the use of molecular genetics. Furthermore, recent *in vivo* and *ex vivo* cell biological techniques, including *in vivo* electroporation, slice culture methods, time-lapse imaging and electron microscopy analyses, have uncovered essential roles for dynamic regulation of cytoskeleton and cell adhesion in neuronal migration. In the locomoting neurons, the formation of dilation/swelling requires proper regulation of microtubules, actin cytoskeleton, and endocytic pathways (Figure 2). Another feature of locomotion, a radial glial fiber-dependent migration, depends on the membrane trafficking-mediated remodeling of the cell adhesion complex (Figure 2). Thus, molecular pieces, identified from molecular genetics and *in vivo* electroporation, begin to take shape. However, the spatio-temporal regulation of these cellular events remains unclear. Furthermore, the dynamic behavior of each endosome in migrating neurons in cortical slices remains to be observed. Continual technological advances in *in vivo* cell biology and related research fields will shed light on unsolved questions to help us better understand the whole picture of cerebral cortical development.

### Conflict of interest statement

The author declares that the research was conducted in the absence of any commercial or financial relationships that could be construed as a potential conflict of interest.
